# Examining Sodium and Potassium Channel Conductances Involved in Hyperexcitability of Chemotherapy-Induced Peripheral Neuropathy: A Mathematical and Cell Culture-Based Study

**DOI:** 10.3389/fncom.2020.564980

**Published:** 2020-10-15

**Authors:** Parul Verma, Muriel Eaton, Achim Kienle, Dietrich Flockerzi, Yang Yang, Doraiswami Ramkrishna

**Affiliations:** ^1^Davidson School of Chemical Engineering, Purdue University, West Lafayette, IN, United States; ^2^Medicinal Chemistry and Molecular Pharmacology, Purdue University, West Lafayette, IN, United States; ^3^Purdue Institute for Integrative Neuroscience, Purdue University, West Lafayette, IN, United States; ^4^Process Synthesis and Dynamics Group, Max Planck Institute for Dynamics of Complex Technical Systems, Magdeburg, Germany; ^5^Chair for Automation/Modeling, Otto von Guericke University, Magdeburg, Germany; ^6^Max Planck Institute for Dynamics of Complex Technical Systems, Magdeburg, Germany; ^7^Otto von Guericke University, Magdeburg, Germany

**Keywords:** chemotherapy-induced peripheral neuropathy, ion channel, bifurcation theory, hyperexcitability, paclitaxel, DRG neuron, multielectrode array

## Abstract

Chemotherapy-induced peripheral neuropathy (CIPN) is a prevalent, painful side effect which arises due to a number of chemotherapy agents. CIPN can have a prolonged effect on quality of life. Chemotherapy treatment is often reduced or stopped altogether because of the severe pain. Currently, there are no FDA-approved treatments for CIPN partially due to its complex pathogenesis in multiple pathways involving a variety of channels, specifically, voltage-gated ion channels. One aspect of neuropathic pain *in vitro* is hyperexcitability in dorsal root ganglia (DRG) peripheral sensory neurons. Our study employs bifurcation theory to investigate the role of voltage-gated ion channels in inducing hyperexcitability as a consequence of spontaneous firing due to the common chemotherapy agent paclitaxel. Our mathematical investigation of a reductionist DRG neuron model comprised of sodium channel Na_v_1.7, sodium channel Na_v_1.8, delayed rectifier potassium channel, A-type transient potassium channel, and a leak channel suggests that Na_v_1.8 and delayed rectifier potassium channel conductances are critical for hyperexcitability of small DRG neurons. Introducing paclitaxel into the model, our bifurcation analysis predicts that hyperexcitability is highest for a medium dose of paclitaxel, which is supported by multi-electrode array (MEA) recordings. Furthermore, our findings using MEA reveal that Na_v_1.8 blocker A-803467 and delayed rectifier potassium enhancer L-alpha-phosphatidyl-D-myo-inositol 4,5-diphosphate, dioctanoyl (PIP_2_) can reduce paclitaxel-induced hyperexcitability of DRG neurons. Our approach can be readily extended and used to investigate various other contributors of hyperexcitability in CIPN.

## 1. Introduction

Chemotherapy-induced peripheral neuropathy (CIPN) is a painful, dose-limiting side effect of chemotherapy cancer treatment that affects more than 85% of patients during treatment (Fallon, 2013) and 60% of patients 3 months post chemotherapy treatment (Seretny et al., 2014). These patients report enduring “pin and needle” paresthesias (i.e., tingling, numbness) in the peripheral nervous system (hands and feet) (Park et al., 2013). The onset of symptoms can range from 1 day to 2 years after treatment and can even persist throughout life, causing a significant decrease in the quality of life of cancer survivors (Cavaletti and Marmiroli, 2010; Speck et al., 2012). Furthermore, CIPN has been shown to result in depression, frustration, and a sense of loss of purpose among these patients (Tofthagen, 2010). To improve quality of life of cancer patients, it is imperative to find CIPN preventive agents. Since there is currently no FDA-approved treatment for CIPN, management of CIPN-induced pain includes many options such as antidepressants, anticonvulsants, anti-inflammatory, and opioid therapies.

Neuropathic pain can be characterized by changes in firing patterns, calcium signaling, mitochondrial dysfunction, axonal transport dysfunction, and inflammation, among others (Meacham et al., 2017). Increases in spontaneous firing has been strongly associated with allodynia but hyperalgesia may be related to reduced threshold of triggered firing or enhanced firing of putative small pain sensing DRG neurons (Devor and Seltzer, 1999). Hyperexcitability from increased spontaneous firing involves voltage-gated sodium channels Na_v_1.6–1.9 (Lou et al., 2005) and potassium channels (Du and Gamper, 2013), among others. Although there is some role of the central nervous system (CNS) in CIPN, treatments targeting CNS-based pain pathways have not been sufficient to reduce CIPN (Chu et al., 2015; Fukuda et al., 2017). Mainly, CIPN has been studied in the dorsal root ganglia (DRG) sensory neurons in the peripheral nervous system (PNS). DRG neurons in the PNS relay sensory signals to neurons in the spinal cord and eventually to the CNS, thus allowing the different subpopulations of DRG neurons to respond to different nociceptive stimuli including mechanical, thermal, and chemical (Dubin and Patapoutian, 2010). DRG neurons are more susceptible to chemotherapy agents than the CNS neurons because DRG neurons do not have an extensive neurovascular barrier to limit drug entry (Lees et al., 2017; Livni et al., 2019). Thus, we chose DRG neurons to be our model system for this study.

A myriad of alterations in various pathways have been linked to CIPN including voltage-gated ion channels, in calcium signaling, in fast axonal transport, and in occurrence of oxidative stress and inflammation (Carozzi et al., 2015). Several chemotherapy agents, such as vincristine, paclitaxel, and oxaliplatin have been suggested to cause CIPN (Carozzi et al., 2015). In this study, we focus on the chemotherapy agent paclitaxel (brand name Taxol). Paclitaxel is a microtubule-binding cancer agent used for several solid tumor cancers such as breast, ovarian, and lung. The conjecture concerning the paclitaxel-induced CIPN (PIPN) mechanism is that it reduces axonal transport of mRNA which can lead to axonal degeneration, alters expression of membrane ion channels, and induces inflammation and oxidative stress (Carozzi et al., 2015). Several agents have been tested in clinical trials, but their ability to prevent PIPN is still unclear (Argyriou et al., 2008). Since it can be difficult to control and examine multiple events in an experimental setting, a mathematical model can be employed to investigate mechanisms by exploring many channels in a controlled manner to observe how the system behavior changes upon any external influences. In this study, we analyze the role of voltage-gated sodium and potassium ion channels in paclitaxel-induced hyperexcitability using mathematical modeling and bifurcation theory, then support our results using *in vitro* recordings. Specifically, our mathematical model is representative of a simplified small DRG neuron model consisting of two sodium channels (Na_v_1.7 and Na_v_1.8), two potassium channels [delayed rectifier (KDR) and A-type transient (KA)], and one leak channel. We included these channels because they were deemed to be most prominent in small DRG neurons (Gold et al., 1996; Sheets et al., 2007; Choi and Waxman, 2011). Here, we report the development of a mathematical modeling-based approach, which is then supported by experimental findings in cell culture-based model of CIPN.

## 2. Materials and Methods

### 2.1. Mathematical Model Description and Equations

The mathematical model here is representative of a small DRG neuron which is simplified to be a single compartment cylindrical model. It consists of sodium channels Na_v_1.7 and Na_v_1.8, delayed rectifier (KDR) and A-type transient (KA) potassium channels, and a leak channel. Equations and parameter values used in this model were from Choi and Waxman (2011). The main equation for membrane voltage is written as:

(1) $$\begin{array}{l} C\frac{\text{d}V}{\text{d}t}=\frac{I_{ext}}{A}-\left(i_{1.7}+i_{1.8}+i_{KDR}+i_{KA}+i_{l}\right), \end{array}$$

where, *I*_*ext*_ is the external applied current, *i*_1.7_, *i*_1.8_, *i*_*KDR*_, *i*_*KA*_, *i*_*l*_ are specific ionic currents due to Na_v_1.7, Na_v_1.8, KDR, KA, and leak channels. Model parameters *C* is the specific membrane capacitance and *A* is the area, and the variables *V* is the membrane voltage and *t* is time. These ionic currents are written as following:

(2) $$\begin{array}{l} i_{1.7}={\bar{g}}_{1.7}m_{1.7}^{3}h_{1.7}s_{1.7}\left(V-E_{Na}\right), \end{array}$$

(3) $$\begin{array}{l} i_{1.8}={\bar{g}}_{1.8}m_{1.8}h_{1.8}\left(V-E_{Na}\right), \end{array}$$

(4) $$\begin{array}{l} i_{KDR}={\bar{g}}_{KDR}n_{KDR}\left(V-E_{K}\right), \end{array}$$

(5) $$\begin{array}{l} i_{KA}={\bar{g}}_{KA}n_{KA}h_{KA}\left(V-E_{K}\right),\text{ and}, \end{array}$$

(6) $$\begin{array}{l} i_{l}={\bar{g}}_{l}\left(V-E_{l}\right), \end{array}$$

where, ${\bar{g}}_{i}$ (*i*=1.7, 1.8, *KDR, KA, l*) are maximal conductances and are constants. Parameters *E*_*Na*_, *E*_*K*_, and *E*_*l*_ are the equilibrium ion potentials. All the activation and inactivation gating variables *x* (*x*=*m*_1.7_, *h*_1.7_, *s*_1.7_, *m*_1.8_, *h*_1.8_, *n*_*KDR*_, *n*_*KA*_, *h*_*KA*_) are written as the following in Hodgkin-Huxley form (Hodgkin and Huxley, 1952):

(7) $$\begin{array}{l} \frac{\text{d}x}{\text{d}t}=\frac{x_{\infty }-x}{\tau_{x}}. \end{array}$$

The expressions of *x*_∞_ and τ_*x*_, and the parameter values are specified below. All the equation forms have been extracted from literature (Choi and Waxman, 2011). The model parameter values and the corresponding references are mentioned in Table 1. Below, we describe the equations for each of the voltage-gated ion channels.

**Table 1:** Model parameter values.

**Parameter**	**Value**	**Units**	**References**
*A* (area)	2168.00	μm^2^	Choi and Waxman, 2011
*C*	0.93	μF/cm^2^	Choi and Waxman, 2011
*E*_*Na*_	67.10	mV	Choi and Waxman, 2011
*E*_*K*_	−84.70	mV	Choi and Waxman, 2011
*E*_*l*_	−58.91	mV	Choi and Waxman, 2011
${\bar{g}}_{1.7}$	18.00	mS/cm^2^	Choi and Waxman, 2011
${\bar{g}}_{1.8}$	7.00	mS/cm^2^	Verma et al., 2020c
${\bar{g}}_{KDR}$	4.78	mS/cm^2^	Choi and Waxman, 2011; Verma et al., 2020c
${\bar{g}}_{KA}$	8.33	mS/cm^2^	Choi and Waxman, 2011; Verma et al., 2020c
${\bar{g}}_{l}$	0.0575	mS/cm^2^	Choi and Waxman, 2011
*k*_0.5_	500	nM	Rowinsky et al., 1999; Rana et al., 2017
*h*_*n*_	2	Unitless	Assumed
${\bar{G}}_{Na,max}$	100	mS/cm^2^	Assumed
${\bar{G}}_{K,min}$	0.1	mS/cm^2^	Assumed

The equations for Na_v_1.7 are written as the following:

(8) $$\begin{array}{l} \alpha_{m_{1.7}}=\frac{15.5}{1+\text{exp}\left(\frac{V-5}{-12.08}\right)}, \end{array}$$

(9) $$\begin{array}{l} \beta_{m_{1.7}}=\frac{35.2}{1+\text{exp}\left(\frac{V+72.7}{16.7}\right)}, \end{array}$$

(10) $$\begin{array}{l} \alpha_{h_{1.7}}=\frac{0.38685}{1+\text{exp}\left(\frac{V+122.35}{15.29}\right)}, \end{array}$$

(11) $$\begin{array}{l} \beta_{h_{1.7}}=-0.00283+\frac{2.00283}{1+\text{exp}\left(\frac{V+5.5266}{-12.70195}\right)}, \end{array}$$

(12) $$\begin{array}{l} \alpha_{s_{1.7}}=0.00003+\frac{0.00092}{1+\text{exp}\left(\frac{V+93.9}{16.6}\right)},\text{ and}, \end{array}$$

(13) $$\begin{array}{l} \beta_{s_{1.7}}=132.05-\frac{132.05}{1+\text{exp}\left(\frac{V-384.9}{28.5}\right)}. \end{array}$$

For *x* = *m*_1.7_, *h*_1.7_, *s*_1.7_,

(14) $$\begin{array}{l} x_{\infty }=\frac{\alpha_{x}\left(V\right)}{\alpha_{x}\left(V\right)+\beta_{x}\left(V\right)},\text{ and}, \end{array}$$

(15) $$\begin{array}{l} \tau_{x}=\frac{1}{\alpha_{x}\left(V\right)+\beta_{x}\left(V\right)}. \end{array}$$

The equations for Na_v_1.7 were extracted from Sheets et al. (2007) and Choi and Waxman (2011). Variables *m*_1.7_ is the activation gating variable, *h*_1.7_ the fast-inactivation gating variable, and *s*_1.7_ the slow-inactivation gating variable.

The equations for Na_v_1.8 are written as the following:

(16) $$\begin{array}{l} \alpha_{m_{1.8}}=2.85-\frac{2.839}{1+\text{exp}\left(\frac{V-1.159}{13.95}\right)}, \end{array}$$

(17) $$\begin{array}{l} \beta_{m_{1.8}}=\frac{7.6205}{1+\text{exp}\left(\frac{V+46.463}{8.8289}\right)}, \end{array}$$

(18) $$\begin{array}{l} m_{1.8_{\infty }}=\frac{\alpha_{m_{1.8}}\left(V\right)}{\alpha_{m_{1.8}}\left(V\right)+\beta_{m_{1.8}}\left(V\right)}, \end{array}$$

(19) $$\begin{array}{l} h_{1.8_{\infty }}=\frac{1}{1+\text{exp}\left(\frac{V+32.2}{4}\right)}, \end{array}$$

(20) $$\begin{array}{l} \tau_{m_{1.8}}=\frac{1}{\alpha_{m_{1.8}}\left(V\right)+\beta_{m_{1.8}}\left(V\right)},\text{ and}, \end{array}$$

(21) $$\begin{array}{l} \tau_{h_{1.8}}=1.218+42.043\times \text{exp}\left(\frac{-{\left(V+38.1\right)}^{2}}{2\times 15.19^{2}}\right). \end{array}$$

The equations for Na_v_1.8 were obtained from Sheets et al. (2007) and Choi and Waxman (2011). Variables *m*_1.8_ is the activation gating variable and *h*_1.8_ is the inactivation gating variable.

The equations for KDR are written as the following:

(22) $$\begin{array}{l} \alpha_{n_{KDR}}=\frac{0.001265\times \left(V+14.273\right)}{1-\text{exp}\left(\frac{V+14.273}{-10}\right)}, \end{array}$$

where, α_*n*_*KDR*__ = 0.001265 × 10 if *V* = −14.273,

(23) $$\begin{array}{l} \beta_{n_{KDR}}=0.125\times \text{exp}\left(\frac{V+55}{-2.5}\right), \end{array}$$

(24) $$\begin{array}{l} n_{KDR_{\infty }}=\frac{1}{1+\text{exp}\left(\frac{-\left(V+14.62\right)}{18.38}\right)},\text{ and}, \end{array}$$

(25) $$\begin{array}{l} \tau_{n_{KDR}}=\frac{1}{\alpha_{n_{KDR}}+\beta_{n_{KDR}}}+1. \end{array}$$

The equations of KDR channel were obtained from Schild et al. (1994). Variable *n*_*KDR*_ is the activation gating variable. Inactivation gating variables are not present.

The equations for KA are written as the following:

(26) $$\begin{array}{l} n_{{KA}_{\infty }}={\left(\frac{1}{1+\text{exp}\left(\frac{-\left(V+5.4\right)}{16.4}\right)}\right)}^{4}, \end{array}$$

(27) $$\begin{array}{l} \tau_{n_{KA}}=0.25+10.04\times \text{exp}\left(\frac{-{\left(V+24.67\right)}^{2}}{2\times 34.8^{2}}\right), \end{array}$$

(28) $$\begin{array}{l} h_{{KA}_{\infty }}=\frac{1}{1+\text{exp}\left(\frac{V+49.9}{4.6}\right)},\text{ and}, \end{array}$$

(29) $$\begin{array}{l} \tau_{h_{KA}}=20+50\times \text{exp}\left(\frac{-{\left(V+40\right)}^{2}}{2\times 40^{2}}\right), \end{array}$$

if τ_*h*_*KA*__(*V*) < 5, τ_*h*_*KA*__(*V*) = 5.

The equations for KA channel were obtained from Sheets et al. (2007). Variables *n*_*KA*_ is the activation gating variable whereas *h*_*KA*_ is the inactivation gating variable. The timescales of all these activation and inactivation variables for each of the aforementioned channels can be found in Verma et al. (2020c).

### 2.2. XPPAUT Settings

All the bifurcation diagrams were primarily generated from XPPAUT (Ermentrout, 2002). Confirmation of the results was done using MATCONT (Dhooge et al., 2008). Moreover, two parameter continuation was performed using MATCONT. All the plots were generated using MATLAB (MATLAB, 2018). The following parameters were used for XPPAUT:

NTST = 100, Method = Stiff, Tolerance = 1e-07, EPSL, EPSU, EPSS = 1e-07, ITMX, ITNW = 20, Dsmin = 1e-05, Dsmax = 0.05. There may be a need to adjust Dsmax to 0.1 and Ds to 0.01. All other settings were same as default.

### 2.3. MATCONT Settings

MATCONT settings were the following: MaxCorrIters = 20, MaxTestIters = 20, FunTolerance = 1e-6, VarTolerance = 1e-6, TestTolerance = 1e-5, MaxStepsize = 0.01. When keeping paclitaxel as the bifurcation parameter, the following settings were changed: MaxStepsize = 0.001, InitStepsize = 0.0001, MinStepsize = 1e-7.

### 2.4. Reagents

Na_v_1.8 blocker A-803467 (200 nM) and KDR enhancer L-alpha-phosphatidyl-D-myo-inositol 4,5-diphosphate, dioctanoyl (PIP_2_, 100 μM) were diluted in NbActiv4 recording media (BrainBits, Springfield, IL, USA). Complete saline solution (CSS) was made from 137 mM NaCl, 5.3 mM KCl, 1 mM MgCl_2_-6H_2_O, 25 mM sorbitol, 10 mM HEPES, and 3 mM CaCl_2_ equilibrated to pH 7.2.

### 2.5. Primary Cell Culture

Dorsal root ganglia (DRG) neurons were extracted from wild-type Sprague-Dawley rat pups 7–14 days old. Age of rats were chosen based on a previous CIPN study (Li et al., 2018) since cultured postnatal DRG neurons share many characteristics of mature DRG neurons (Melli and Hake, 2009). Animals were maintained in the norovirus-negative facility of the Centrally Managed Animal Facilities at Purdue University. They were housed in at a constant temperature and humidity on a 12:12 light-dark cycle (lights on 06:00–18:00) with ad lib access to food and water according to as approved by the Purdue Animal Care and Use Committee and the Institutional Animal Care and Use Committee (IACUC) and was conducted in accordance with the National Institutes of Health Guide for the Care and Use of Laboratory Animals. Pups were placed on a paper towel on ice for 2 min before decapitation. The spinal cord was extracted and DRG neurons were dissected and placed in complete saline solution (CSS). Cells were prepared by centrifuging at 2.5×g for 30 s then adding 1.5 mg/mL of collagenase A in CSS with 0.05 mM EDTA. After rotating in the 37°C incubator for 20 min, cells were spun at 2.5×g for 30 s. 1.5 mg/mL collagenase D and 30U papain in CSS were added and then placed in the incubator rotator for 20 min. Cells were spun at 2.5×g for 3 min. Primary DRG neurons were triturated in 1 mL of 0.15% trypsin inhibitor and 0.15% bovine serum albumin (BSA) in Dulbecco's Modification of Eagle's Medium (DMEM) medium with 10% fetal bovine serum (FBS) (Corning, Corning, NY, USA) and then spun again at 2.5×g for 3 min then filtered through a 40μM filter before seeding on a plate coated with laminin and poly-d-lysine. DRG neurons from one pup were divided into six wells. Culture media was changed after 2 days to NbActiv4 recording media (BrainBits, Springfield, IL, USA). Four days after seeding, culture was recorded.

### 2.6. Micro/Multielectrode Array (MEA)

Firing properties were recorded using a Maestro Pro (Axion BioSystems, Atlanta, GA, USA). Twelve well plates of 64 electrodes per well were used for culture. Plates were coated the day of seeding by incubating with poly-d-lysine for 2 h, washing with sterile milli-q water three times, then incubating with laminin for 1 h. All recordings were at 37°C. The plate was recorded before treatment and 24 h after treatment. Then 200 μL of 1.0 μM capsaicin was added and recorded for 2 min as a positive control. Analysis was performed using the manufacturer's software, Axion BioSystems Integrated Studio (AxIS) and NeuroMetric Tool. An electrode (*n* = 1) was considered active if there were more than two action potentials in the baseline, response to buffer, or response to capsaicin. Mean firing rate (Hz) was calculated for active electrodes. Chili pepper compound capsaicin was added into the system to trigger a sensory response. Fold change was calculated from natural logarithm of firing rate in line with literature (Atmaramani et al., 2020; Negri et al., 2020). Percent change described in Table 2 is the number of spontaneous firing neurons after treatment minus the number before treatment over number before treatment. Recordings were compiled from different cultures extracted from different animals on different days. Although one electrode may detect more than one neuron, this situation is unlikely to occur to a notable level, due to low basal firing and low plating density of primary DRG sensory neurons in the MEA experiment (Yang et al., 2016, 2018).

**Table 2:** Number of firing neurons.

**Treatment**	**Paclitaxel (nM)**	**Fold change**	**Percent change %**
Media	None	0.94	−3.64
Pax	250	1.42	21.09
Na_v_1.8-	250	1.27	11.83
KDR+	250	1.28	5.64

*Pax, Paclitaxel; Na_v_1.8-, Na_v_1.8 blocker; KDR+, KDR enhancer*.

### 2.7. Statistical Analyses

A Shapiro–Wilk test determined that the data was not normally-distributed so Krushal–Wallis and Mann–Whitney *U*-tests were used to determine statistical significance of treatment differences. MEA results are presented as mean (median, range, sample size, *p*-value). One-sided chi-square test was used to analyze the increase in firing rate from baseline to treatment. Also, this test was used to analyze the change in qualitative behavior of neurons by comparing the number of neurons with a firing rate of zero in the baseline and with a > 0 firing rate after treatment. *P*-values were reported at *p ≤ 0.05, **p ≤ 0.01, and ***p ≤ 0.001. GraphPad Prism 8.3.0 was used to determine statistical significance.

## 3. Results

### 3.1. Model Simulations

One indicator of peripheral neuropathy is spontaneous firing which implies repetitive firing of action potentials for *I*_*ext*_ = 0. In this work, we explore the parameters that can lead to spontaneous firing and can be potentially impacted by paclitaxel. We vary the maximal ion conductances to explore whether they can induce spontaneous firing since current literature indicates that paclitaxel can manipulate the expression of voltage-gated ion channels (Zhang and Dougherty, 2014). A bifurcation analysis of this model with *I*_*ext*_ as the bifurcation parameter can be found elsewhere (Verma et al., 2020c).

**Figure 1:**
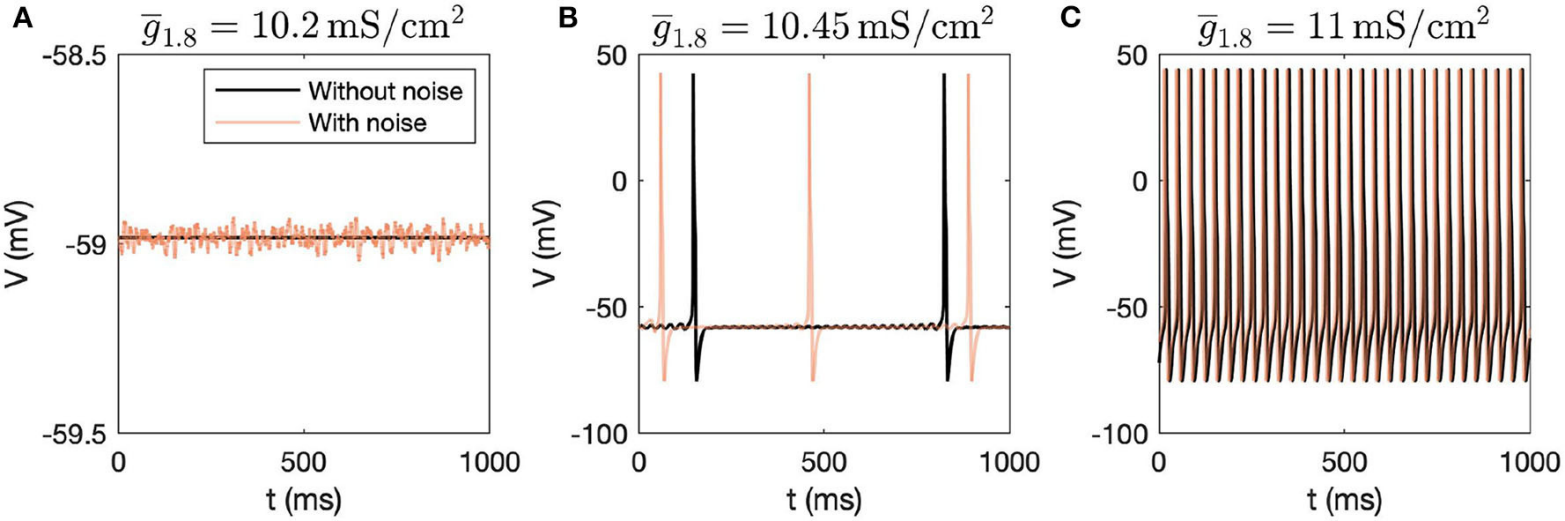
Dynamic simulations obtained by varying ${\bar{g}}_{1.8}$, with and without introducing noise in the system. **(A)** Steady state is observed for ${\bar{g}}_{1.8}\;$= 10.2mS/cm^2^, **(B)** MMOs are observed for ${\bar{g}}_{1.8}\;$= 10.45mS/cm^2^, and **(C)** Continuous firing of action potentials is observed for ${\bar{g}}_{1.8}\;$= 11mS/cm^2^.

First, we performed dynamic simulations for different parameter values. The initial conditions correspond to the stable steady state solution obtained for the parameter values mentioned in Table 1. Figure 1 demonstrates how the voltage dynamics vary upon increasing ${\bar{g}}_{1.8}$ (${\bar{g}}_{1.8}$ was chosen for illustration purposes), without and with noise (following Rho and Prescott, 2012 for including noise). Without noise, for low values of ${\bar{g}}_{1.8}$, the system settles down to a steady state, shown in Figure 1A. For higher values of ${\bar{g}}_{1.8}$, mixed-mode oscillations (MMOs) are observed, shown in Figure 1B. MMOs consist of both small amplitude (subthreshold) and large amplitude (action potential) oscillations. For higher values, continuous firing of action potentials is observed, shown in Figure 1C. In the next section, we focus on the switch from steady state to continuous firing by treating different channel conductances as bifurcation parameters. We have majorly focused on the model without noise and have only demonstrated how simulations vary upon introducing noise. Mixed-mode oscillations and the impact of noise were investigated in detail in another work (Verma et al., 2020b).

### 3.2. Bifurcation Analysis

XPPAUT is used to perform preliminary bifurcation analysis, and confirmation of results is done using MATCONT. Parameter settings for XPPAUT and MATCONT are mentioned in section 2. As mentioned before, bifurcation diagrams are generated by setting the maximal conductance as the bifurcation parameter, one by one, for each voltage-gated ion channel involved in this model (Na_v_1.7, Na_v_1.7, KDR, and KA).

#### 3.2.1. One-Parameter Continuation

**Figure 2:**
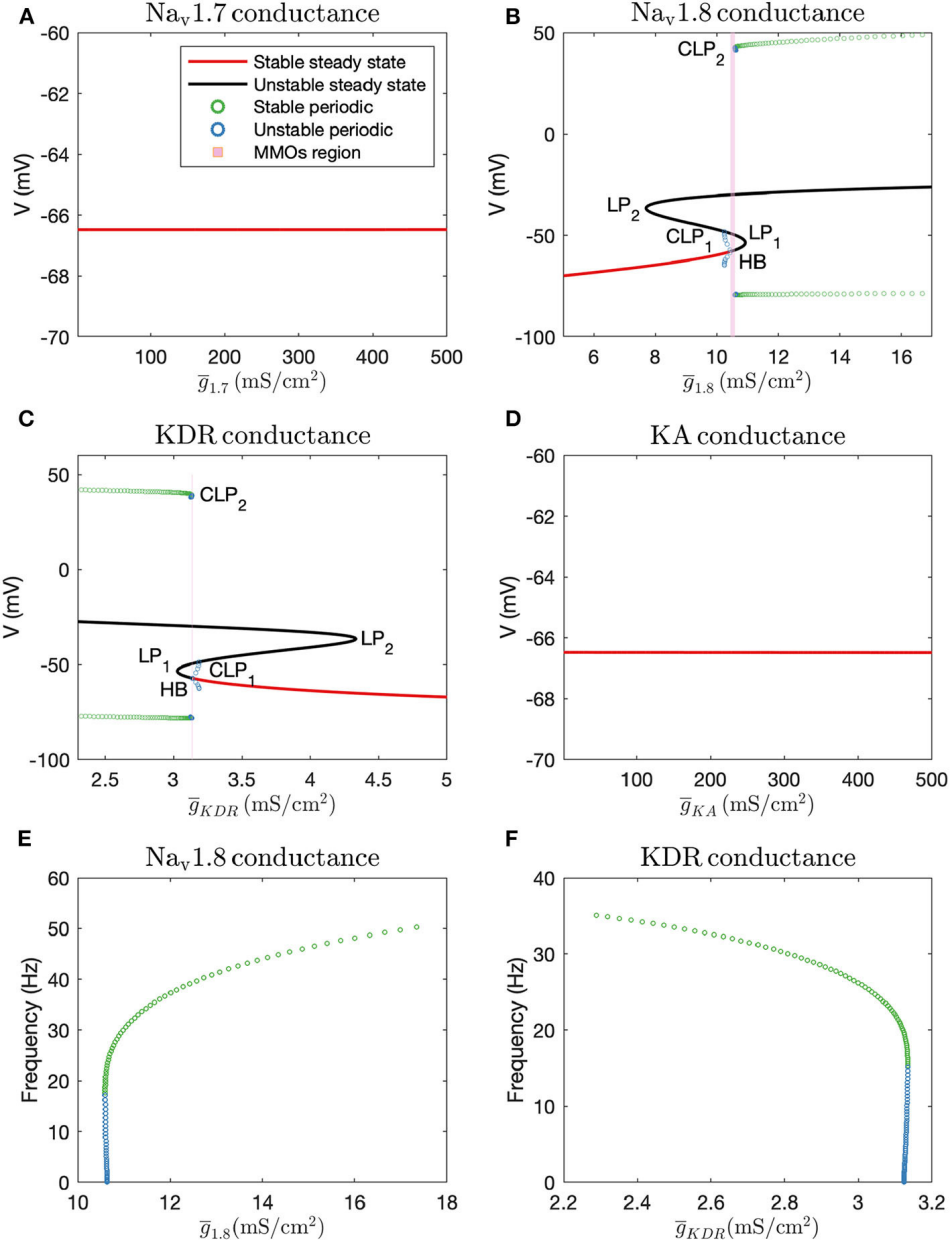
Bifurcation diagrams obtained by keeping **(A)**: ${\bar{g}}_{1.7}$, **(B)**: ${\bar{g}}_{1.8}$, **(C)**: ${\bar{g}}_{KDR}$ (note that there is a thin gap between HB and CLP_2_ where MMOs are observed, which may not be visible without zooming in the figure), and **(D)**: ${\bar{g}}_{KA}$ as the bifurcation parameters. HB: Supcritical Hopf bifurcation point, LP_1_ and LP_2_: limit points, CLP_1_ and CLP_2_: cyclic limit points. Frequency vs. maximal conductance obtained in the periodic firing regime with **(E)**: ${\bar{g}}_{1.8}$ and **(F)**: ${\bar{g}}_{KDR}$ as the bifurcation parameters. The frequency of firing increases with ${\bar{g}}_{1.8}$ and decreases with ${\bar{g}}_{KDR}$. The frequency of unstable periodic solutions tends toward zero, implying that the unstable branch is ending in a period-infinity solution.

Firstly, we performed one-parameter continuations to find bifurcation points which could separate steady state from MMOs, and MMOs from continuous firing of action potentials. A partial bifurcation diagram is shown in Figure 2. As seen in Figures 2A,D, no bifurcation points are generated upon varying ${\bar{g}}_{1.7}$ and ${\bar{g}}_{KA}$. A single red line, representing stable steady state solutions, is observed. On the contrary, bifurcation points are observed in Figures 2B,C. In Figure 2B, a subcritical Hopf bifurcation point (HB) is detected upon increasing ${\bar{g}}_{1.8}$. Beyond this point, steady state solutions become unstable, shown by the black branch. Two turning points/limit points (LP_1_, LP_2_) are also detected on the black branch of unstable steady state solutions. Since the Hopf bifurcation point is subcritical, unstable periodic solutions emanate from it, as shown by the blue branch. This branch first turns at a cyclic limit point (CLP_1_) and then meets the unstable steady state branch, indicating a homoclinic orbit. This turning at CLP_1_ is not obvious from the figure, however, it can be observed upon zooming into the branch. Upon moving in the backward direction starting from a large value of ${\bar{g}}_{1.8}$ resulting in stable periodic solutions, a stable periodic solution branch is generated, indicated in green, finally leading to a cyclic limit point (CLP_2_) beyond which the periodic solutions become unstable, indicated in blue. This unstable periodic branch abruptly ends due to the period of the branch increasing substantially, indicating that it may tend toward a period-infinity solution, as shown in Figure 2E. The stable periodic solution branch indicates the spontaneous firing parameter regime. The frequency of spontaneous firing increase with ${\bar{g}}_{1.8}$, as shown in Figure 2E. MMOs are found in some region between the Hopf bifurcation point (HB) and the cyclic limit point (CLP_2_), shown by the shaded pink region. Moreover, as was observed in Rho and Prescott (2012), we noted that the parameter range of MMOs will become wider upon introducing noise in the model. We have not explored the bifurcations in the MMOs regime; however, a detailed discussion for a similar situation is given in Verma et al. (2020b).

A similar, although horizontally flipped, bifurcation diagram is generated with ${\bar{g}}_{KDR}$ as the bifurcation parameter, shown in Figure 2C. Upon decreasing ${\bar{g}}_{KDR}$, a subcritical Hopf bifurcation point (HB) is detected with unstable periodic solutions emanating from it. These unstable periodic solutions also intersect the unstable steady state branch, indicating a homoclinic orbit. Similar to Figure 2B, a stable periodic solution branch (indicated by green circles) is detected as well which becomes unstable after a cyclic limit point (CLP_2_). As shown in Figure 2F, the unstable periodic solution again seems to tend toward a period-infinity solution. Moreover, the frequency of spontaneous firing decreases with increase in ${\bar{g}}_{KDR}$.

These bifurcation diagrams indicate that manipulating ${\bar{g}}_{1.8}$ or ${\bar{g}}_{KDR}$ can induce spontaneous firing, while manipulating ${\bar{g}}_{1.7}$ or ${\bar{g}}_{KA}$ will not decrease hyperexcitability. Therefore, to reverse hyperexcitability, Na_v_1.8 and KDR channels should be targeted. We targeted these channels in the case of paclitaxel-induced hyperexcitability, in a primary rodent DRG neuron culture, the results of which are described in the section 3.4.

#### 3.2.2. Two-Parameter Continuation

**Figure 3:**
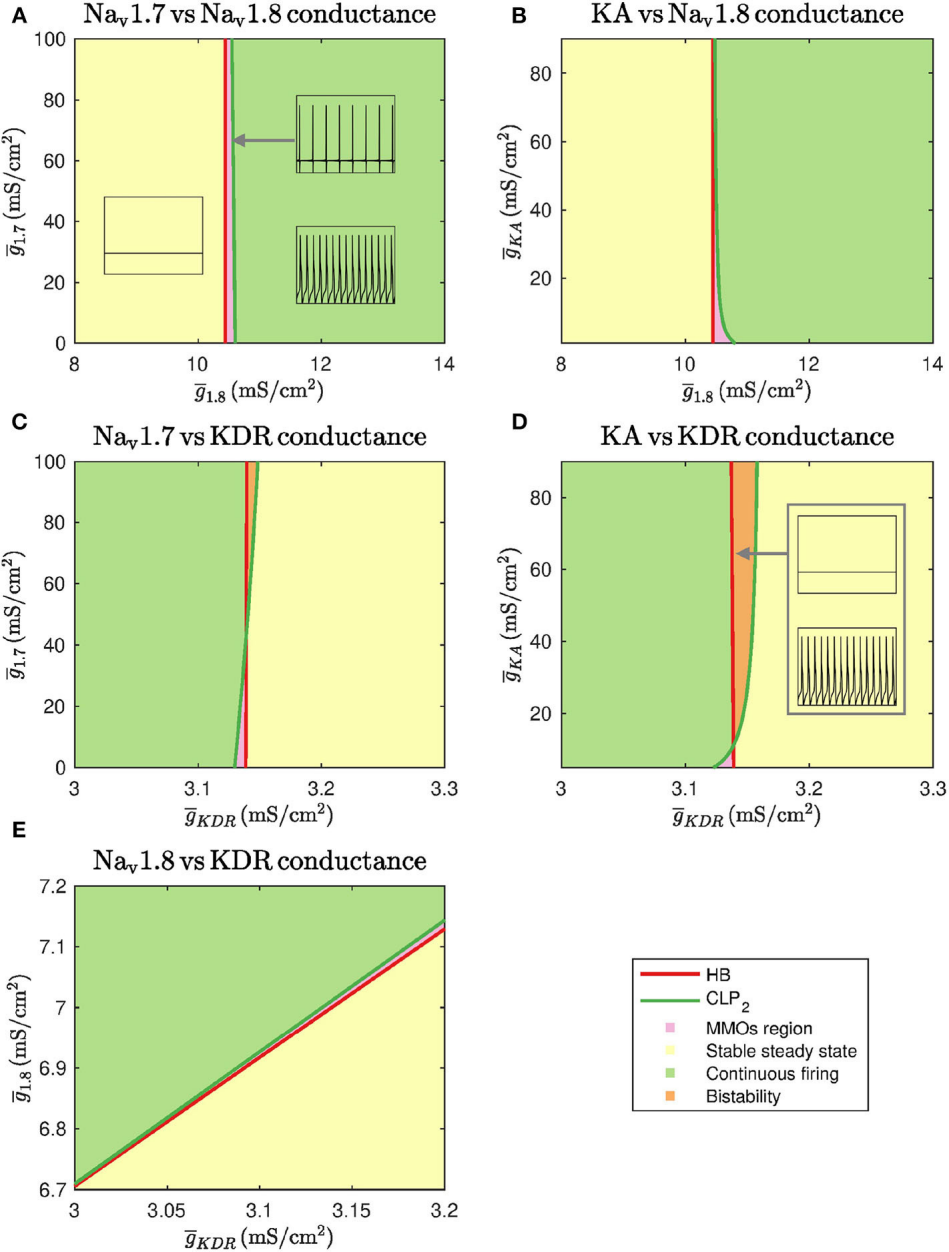
Two parameter continuations performed for the Hopf bifurcation point HB and cyclic limit point CLP_2_. **(A)** Continuation plot for ${\bar{g}}_{1.7}$ vs. ${\bar{g}}_{1.8}$ show that the bifurcation points generated by keeping ${\bar{g}}_{1.8}$ as the bifurcation parameter do not shift upon varying ${\bar{g}}_{1.7}$. There is a range of ${\bar{g}}_{1.8}$ where MMOs can be observed. **(B)** The HB of ${\bar{g}}_{1.8}$ bifurcation diagram do not shift upon varying ${\bar{g}}_{KA}$. CLP_2_ shift rightwards upon decreasing ${\bar{g}}_{KA}$. This implies that the MMOs region will be wider in this case. **(C)** Bifurcation points of ${\bar{g}}_{KDR}$ do not shift upon varying ${\bar{g}}_{1.7}$. For a narrow range of ${\bar{g}}_{KDR}$, MMOs and bistability between stable steady state and continuous firing are observed. **(D)** The HB of ${\bar{g}}_{KDR}$ do not shift upon varying ${\bar{g}}_{KA}$. CLP_2_ shifts leftwards upon decreasing ${\bar{g}}_{KA}$. This implies that the MMOs region will become narrower in this case. A region of bistability is also observed for a narrow range of ${\bar{g}}_{KDR}$. **(E)** A linear combinational effect is seen between ${\bar{g}}_{1.8}$ and ${\bar{g}}_{KDR}$. Note that the thin gap between stable steady state and continuous firing regimes is the MMOs region.

We also performed two-parameter continuation in order to explore the combinational effects of these conductances. To this end, we observed changes in the detected bifurcation points upon altering another maximal conductance. In particular, we performed continuation of HB and CLP_2_. These results are shown in Figure 3.

As seen in Figures 3A,B, ${\bar{g}}_{1.7}$ and ${\bar{g}}_{KA}$ do not substantially impact the bifurcation points even in combination with ${\bar{g}}_{1.8}$. Decreasing ${\bar{g}}_{KA}$ can shift the cyclic limit point of ${\bar{g}}_{1.8}$ to the right, as seen in Figure 3B, which implies that the MMOs regime will become wider. In both the cases, the bifurcation points vary within a narrow range of ${\bar{g}}_{1.8}$. Similarly, Figures 3C,D show that ${\bar{g}}_{1.7}$ and ${\bar{g}}_{KA}$ do not impact the bifurcation points substantially even in combination with ${\bar{g}}_{KDR}$. In these two cases, an increase in ${\bar{g}}_{1.7}$ and ${\bar{g}}_{KA}$ can lead to a region of bistability where stable steady state and continuous firing of action potentials solutions coexist. However, the bifurcation points only vary within a narrow range of ${\bar{g}}_{KDR}$. Upon varying ${\bar{g}}_{1.8}$ and ${\bar{g}}_{KDR}$ together, the bifurcation points vary linearly, as shown in Figure 3E. This indicates that decreasing ${\bar{g}}_{1.8}$ and increasing ${\bar{g}}_{KDR}$ can eliminate spontaneous firing.

### 3.3. Effect of Paclitaxel

Current literature suggests that paclitaxel can impact gene expression of various voltage-gated ion channels (Zhang and Dougherty, 2014; Aromolaran and Goldstein, 2017). However, it is not known whether the impact is direct or indirect. Evidence indicates that paclitaxel impacts inflammatory cytokines, which can subsequently manipulate the ion channels (Aromolaran and Goldstein, 2017). For example, these inflammatory signals can increase sodium current (Wang et al., 2012). Moreover, a sigmoidal shaped dose-dependent relation is observed between paclitaxel and macrophage IL-12, as seen in Figure 3 in Mullins et al. (1999). Based on this evidence, we assumed a Hill's kinetics type relation between paclitaxel and ion channel maximal conductances. Hill's kinetics are widely used to model dose-response curves. We assume that the conductances will vary as a function of paclitaxel dosage. Moreover, we assume that paclitaxel will lead to an increase in maximal conductance of both the sodium channels, while it would lead to a decrease in maximal conductance of both the potassium channels since these cases would lead to spontaneous firing. We assume that all the conductances are impacted even though it may not lead to a change in spontaneous firing, as shown in the previous section. Therefore, we consider the following relationships:

(30) $$\begin{array}{l} {\bar{g}}_{1.7,new}={\bar{g}}_{1.7}+\left({\bar{G}}_{Na,max}-{\bar{g}}_{1.7}\right)\frac{{\left[\text{P}\right]}^{h_{n}}}{{\left[\text{P}\right]}^{h_{n}}+k_{0.5}^{h_{n}}}, \end{array}$$

(31) $$\begin{array}{l} {\bar{g}}_{1.8,new}={\bar{g}}_{1.8}+\left({\bar{G}}_{Na,max}-{\bar{g}}_{1.8}\right)\frac{{\left[\text{P}\right]}^{h_{n}}}{{\left[\text{P}\right]}^{h_{n}}+k_{0.5}^{h_{n}}}, \end{array}$$

(32) $$\begin{array}{l} {\bar{g}}_{KDR,new}={\bar{g}}_{KDR}+\left({\bar{G}}_{K,min}-{\bar{g}}_{KDR}\right)\frac{{\left[\text{P}\right]}^{h_{n}}}{{\left[\text{P}\right]}^{h_{n}}+k_{0.5}^{h_{n}}}, \end{array}$$

(33) $$\begin{array}{l} {\bar{g}}_{KA,new}={\bar{g}}_{KA}+\left({\bar{G}}_{K,min}-{\bar{g}}_{KA}\right)\frac{{\left[\text{P}\right]}^{h_{n}}}{{\left[\text{P}\right]}^{h_{n}}+k_{0.5}^{h_{n}}}, \end{array}$$

where, *h*_*n*_ is Hill's coefficient, [P] is the paclitaxel concentration (in nM), *k*_0.5_ is the half maximal effective concentration, ${\bar{g}}_{i}$ (*i*=1.7, 1.8, *KDR, KA*) is the original maximal conductance value, ${\bar{g}}_{i,new}$ is the updated maximal conductance value from the above equation. The upper and lower limit of maximal conductances is represented by ${\bar{G}}_{Na,max}$ and ${\bar{G}}_{K,min}$, respectively.

**Figure 4:**
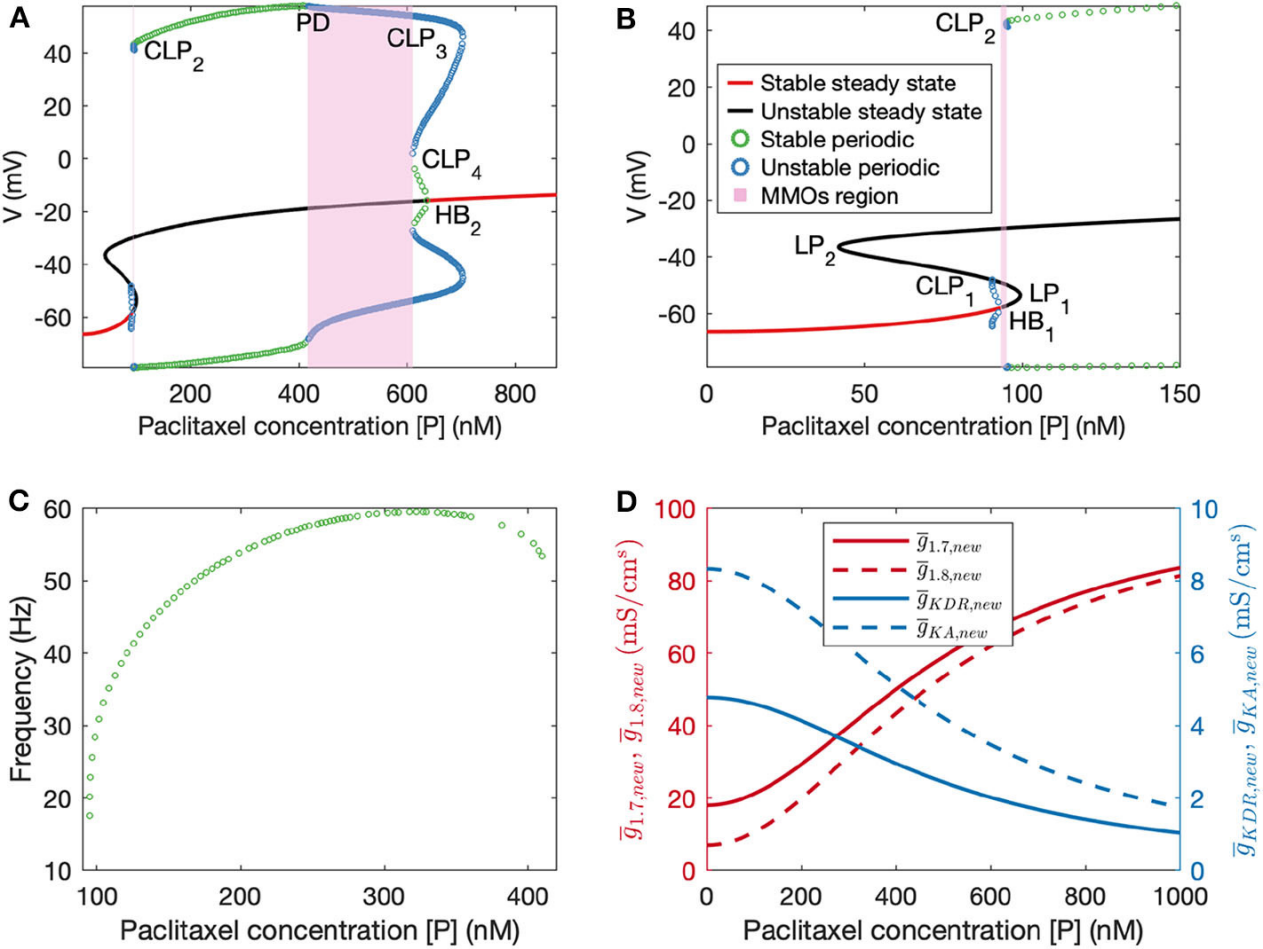
**(A)** Bifurcation diagram obtained by treating paclitaxel concentration as the bifurcation parameter. **(B)** A zoomed in version of the bifurcation diagram in **(A)**. HB_1_: subcritical Hopf bifurcation point, HB_2_: supercritical Hopf bifurcation point, LP_1_ and LP_2_: limit points, CLP_1_, CLP_2_, CLP_3_, and CLP_4_: cyclic limit points, PD: periodic doubling bifurcation point. **(C)** Frequency plot for the stable periodic firing region. Frequency first increases and then decreases upon increasing paclitaxel concentration. Left and right end points of this curve refer to CLP_2_ and PD, respectively. **(D)** Plots of updated maximal conductances vs. paclitaxel concentration for the model parameters used.

Depending on ${\bar{G}}_{j}$ (*j*=*Na, max, K, min*) and *h*_*n*_, the relationship between [P] and ${\bar{g}}_{i,new}$ (*i*=1.7, 1.8, *KDR, KA*) will vary, as shown in Supplementary Figures 1A–D. Increasing or decreasing ${\bar{G}}_{j}$ increases or decreases the maximal conductance parameter (${\bar{g}}_{i,new}$) range covered upon varying [P], respectively. Large *h*_*n*_ creates sigmoidal curves. Decreasing *h*_*n*_ makes the curve seem exponential. In the remaining text, representative values for ${\bar{G}}_{j}$ and *h*_*n*_ were taken as listed in Table 1 and the relationship is demonstrated in Figure 4D. In Supplementary Figure 2, a sensitivity analysis with respect to these parameters was conducted, which shows that the qualitative behavior does not vary much upon varying these parameter values.

Following this, we performed numerical bifurcation analysis with paclitaxel concentration [P] as the bifurcation parameter. A partial bifurcation diagram is shown in Figure 4A which reveals that upon stable steady state solution continuation, a subcritical Hopf bifurcation point (HB_1_) is found beyond which the solutions become unstable. Unstable periodic solutions emanate from this Hopf bifurcation point. Upon continuation of the unstable steady state solution branch in black, a supercritical Hopf bifurcation point (HB_2_) is found, beyond which the steady state solutions become stable again. Stable periodic solutions (indicated in green) emanate from this point. The paclitaxel-interval between these two Hopf bifurcation points constitutes the spontaneous firing regime. The subinterval where the periodic and the steady state branch are unstable (between PD and CLP_4_) corresponds to the MMOs regime. MMOs of this model have been studied in some detail in Verma et al. (2020b); a detailed investigation of the MMOs shown in Figure 4A was beyond the scope of this work. Stable periodic solutions with a small amplitude arise from the supercritical Hopf bifurcation point which turn unstable after a cyclic limit point (CLP_4_). The unstable periodic solutions finally become stable after a subcritical period doubling bifurcation point (PD) when going in the direction of decreasing [P]. The stable periodic solutions become unstable again after a cyclic limit point (CLP_2_).

The frequency of firing in the first stable periodic solutions regime (between CLP_2_ and PD) is shown in Figure 4C. It is shown that upon increasing paclitaxel concentration, frequency of firing first increases, and then decreases after reaching a maximum firing rate. Beyond the PD point, the frequency of firing decreases further since the solutions are of MMOs type.

### 3.4. Experimental Validation Results

A high-throughput way to continuously record neuronal firing patterns is to use multielectrode array (also referred to as microelectrode array, MEA). This system is capable of recording of extracellular voltage potentials with millisecond temporal resolution of neurons in cultures grown on a 768 array of electrodes up to 96 well format, which makes it high-throughput.

**Figure 5:**
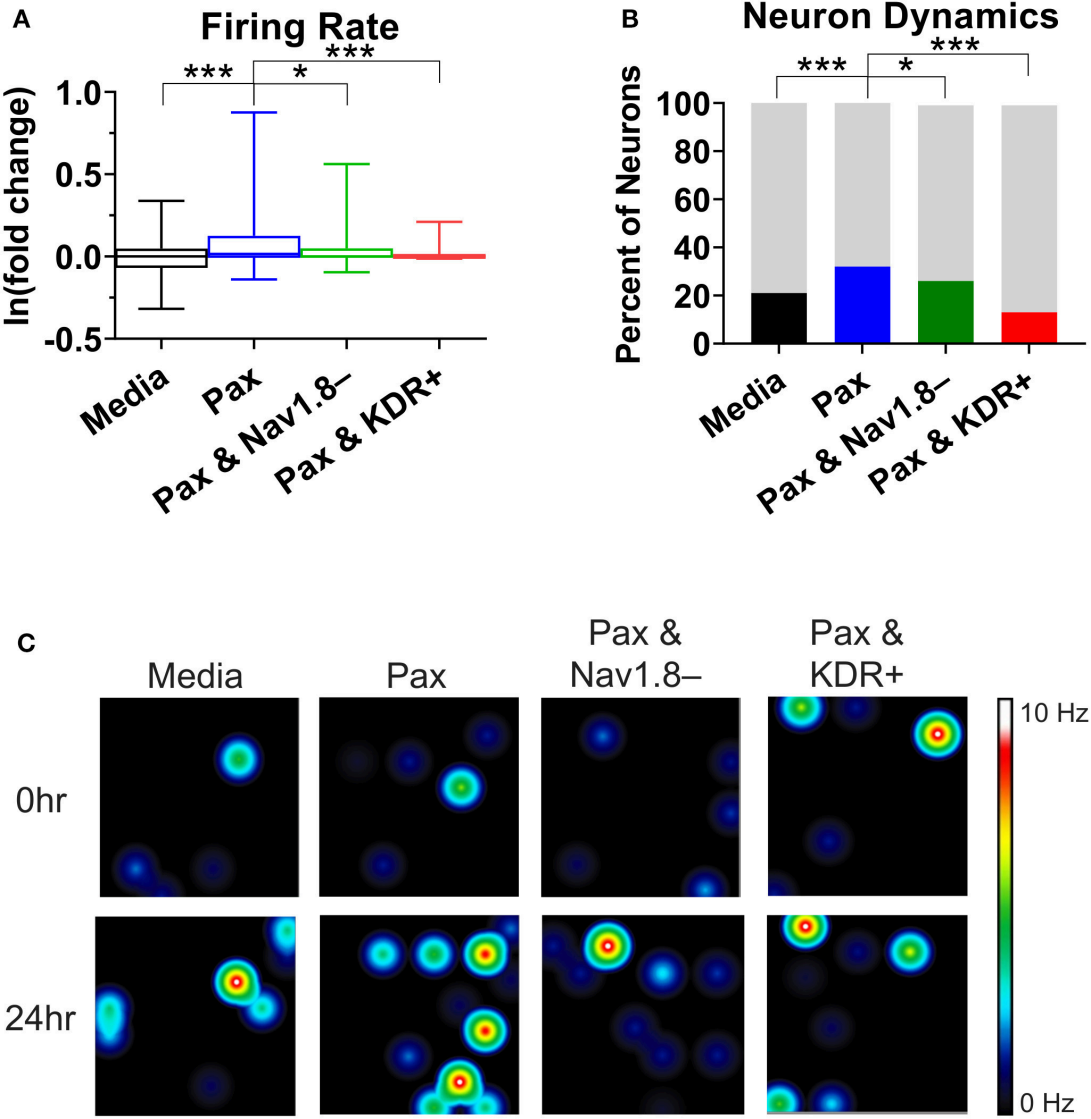
Analysis of multielectrode array (MEA) recording summary shows amelioration of hyperexcitability after treatment of A-803467 (Na_v_1.8 blocker) and PIP_2_ (KDR enhancer). **(A)** Firing rate reveals a significant increase in paclitaxel firing from media control (*p* < 0.0001), a significant decrease from paclitaxel when A-803467 or PIP_2_ are administered concurrently with paclitaxel (*p* = 0.0451 and *p* < 0.0001, respectively) (*n* = 275 for media, 442 for paclitaxel, 338 for paclitaxel and A-803467, 266 for paclitaxel and PIP_2_). Box and whisker plots include a box at the 25^th^ and 75^th^ percentiles, whiskers extend to the 10^th^ and 90^th^ percentiles, and the line is at the median. **(B)** Dynamic neurons are described as neurons that had a firing rate of zero in the baseline with an increased firing rate after treatment. Bottom bar (black or colored) is the percent of neurons that had a firing rate of zero in baseline with an increased firing rate after treatment. Top bar (gray) is the percent neurons that did not fall in this category. **(C)** Heatmap of representative MEA recordings with firing frequency of each active electrode color-coded with warm colors (red, orange, yellow) representing high firing frequency (white = 10 Hz) and cool colors (green, blue) representing low firing frequency (black = 0 Hz). Each circle represents a spontaneously firing neuron within the 8 × 8 electrode array. Top row is baseline at time zero before treatment is added. Bottom row is 24 h after treatment was added. Asterisks denote statistical significance from Mann–Whitney *U*-test **(A)** or chi-square **(B)** (*P < 0.05, ***P < 0.001).

To support the findings of the mathematical modeling of the effect of paclitaxel dose on hyperexcitability, we measured the firing rate for different doses of paclitaxel. Low doses (10 nM) and high doses (1 μM) of 24-h paclitaxel administration caused lower firing rate than 250 nM paclitaxel as expected (see Supplementary Statistics File). Thus, we decided to use 250 nM for the dose of the subsequent experiments. Na_v_1.8 blocker A-803467 and KDR enhancer L-alpha-phosphatidyl-D-myo-inositol 4,5-diphosphate, dioctanoyl (PIP_2_) when administered separately together with paclitaxel reduces the number of spontaneous firing neurons (Table 2) and the firing rate (Figure 5A). Similarly, the representative heat maps of firing frequency reveals the same trend qualitatively (Figure 5C). Specifically, the natural log of firing rate fold change (treatment/baseline) increases with paclitaxel addition to a mean of 0.15 (median = 0.016, range = −2.97 to 3.26, *n* = 442, *p* < 0.0001 compared to media). Na_v_1.8 blocker A-803467 decreases the natural log of firing rate to a mean of 0.08 (median = 0.001, range = −2.40 to 2.50, *n* = 338, *p* = 0.0451 compared to paclitaxel). Similarly, KDR enhancer PIP_2_ reduced paclitaxel-induced hyperexcitability to 0.08 (median = 0.00, range = −3.34 to 2.86, *n* = 266, *p* < 0.0001 compared to paclitaxel).

A chi-square analysis revealed that paclitaxel-treated wells had more neurons whose firing rate increased from baseline compared to the media control (χ^2^ = 22.47, *z* = 4.74, *p* < 0.0001). Wells treated with Na_v_1.8 blocker A-803467 had less neurons whose firing rate increased from baseline as compared to those with only paclitaxel treatment (χ^2^ = 5.85, *z* = 2.42, *p* = 0.0078). Similarly, wells treated with KDR enhancer PIP_2_ had less neurons whose firing rate increased from baseline compared to those with only paclitaxel treatment (χ^2^ = 86.55, *z* = 9.30, *p* < 0.0001). We also compared the MEA results with our bifurcation results from Figures 2, 4 which showed that increasing ${\bar{g}}_{1.8}$ or [P] to some extent, or decreasing ${\bar{g}}_{KDR}$ can lead to spontaneous firing. To this end, we analyzed the number of neurons that did not fire in the baseline, but fired in the treatment, which we considered as a change in the qualitative behavior of the dynamics of these neurons (Figure 5B). We refer to these neurons as dynamic neurons. Paclitaxel-treated wells had an increased number of dynamic neurons compared to the media control (χ^2^ = 10.28, *z* = 3.21, *p* = 0.0007). The Na_v_1.8 blocker A-803467 treated group had a decrease in dynamic neurons compared to paclitaxel treated group (χ^2^ = 3.46, *z* = 1.86, *p* = 0.03) as did the KDR enhancer PIP_2_ treated group (χ^2^ = 31.77, *z* = 5.64, *p* < 0.0001). These results support the hypothesis that blocking Na_v_1.8 or enhancing KDR can reduce paclitaxel-induced hyperexcitability.

## 4. Discussion

CIPN is a debilitating experience for cancer patients with no current established methods of preventing or treating it due to minimal understanding of its pathophysiology (Park et al., 2013). In this paper, we apply a mathematical approach using numerical bifurcation theory to understand the role of sodium channel Na_v_1.7, sodium channel Na_v_1.8, delayed rectifier potassium channel, and A-type transient potassium channel in CIPN in a putative small DRG neuron model. Maximal conductances were kept as bifurcation parameters to identify which of these channels can induce spontaneous firing (one of many indicators of peripheral neuropathy). We further used MEA experiments to support our findings.

Using bifurcation theory, we found that, increasing ${\bar{g}}_{1.8}$ and decreasing ${\bar{g}}_{KDR}$ can induce spontaneous firing (see Figure 2) in a simplified DRG neuron model. The effect may be aggravated in combination, as seen from the two parameter plot in Figure 3E. Our results indicate that a Na_v_1.8 blocker should reduce spontaneous firing which supports the role of Na_v_1.8 in contributing to the increased excitability in peripheral neuropathy (Xiao et al., 2019; Zhang et al., 2019). The significance of blocking Na_v_1.8 in PIPN was also observed in our MEA experiment in which Na_v_1.8 blocker A-803467 had a neuroprotective effect on paclitaxel-induced increased firing rate when administered at the same time as paclitaxel in primary DRG neuron culture (Figure 5). Similarly, KDR was indicated in our model and MEA findings to be involved with hyperexcitability which is supported by literature (Du and Gamper, 2013). Although these results are for specific dosages of A-803467 and PIP_2_ to observe the change in excitability, this electrophysiology data supports the trends found in the mathematical model analysis. Depending on the amount of A-803467 and PIP_2_, hyperexcitability should change. Interestingly, Na_v_1.8 and KDR channels were found to be sensitive even with *I*_*ext*_ as the primary bifurcation parameter (Verma et al., 2020b). It will be of interest to investigate their protective effects on CIPN due to other chemotherapy agents such as vincristine and oxaliplatin, as well.

We assume a Hill's type kinetics for the effect of paclitaxel on the ion channels. Based on this, a partial bifurcation diagram is generated, treating paclitaxel concentration as the bifurcation parameter. This bifurcation diagram indicates that spontaneous firing should arise for a mid-range of paclitaxel dosage. Moreover, firing rate should first increase and then decrease, seen from the frequency diagram in Figure 4C. A similar trend is seen in the MEA recordings, shown in the Supplementary Statistics File. Firing rate is larger for the middle value of paclitaxel concentration in the range considered here. The frequency diagram (Figure 4C) may not reflect the actual reason for this trend. It may be that the cells died at a higher concentration, or they may not fire because of manipulation of the ion channels as shown by a mathematical relationship with paclitaxel. Further investigation is required to establish the reason behind this observation with certainty.

The parameters for the relationship between paclitaxel and ion channel maximal conductances are also assumed (see Table 1). In the Supplementary Figure 2, we have shown how the behavior of the model varies upon changing these parameters. Upon varying *h*_*n*_, ${\bar{G}}_{Na,max}$, or ${\bar{G}}_{K,min}$, the qualitative behavior of the diagram does not change for the range of parameters considered here; stable steady states, MMOs, and continuous firing regimes are observed. Assuming that Hill's kinetics is a reasonable relationship between paclitaxel and ion channel conductance, defined upper and lower limits of how much the conductances can vary may exist. These parameters can be estimated by recording currents due to each of these ion channels for different doses of paclitaxel, using patch-clamping experiments. Lastly, the value of *k*_0.5_ we used in our mathematical model (500 nM) is different from the paclitaxel dose amount used for the MEA experiment (250 nM). This is because we estimated the value from literature which was based on the IC50 value for iPSC-derived human neurons and clinical data based on paclitaxel's toxicity on cancer cells (Rowinsky et al., 1999; Rana et al., 2017). However, as seen in Figure 4C, maximum spontaneous firing is observed around 300 nM. Since we only intend to perform a qualitative comparison between the bifurcation diagram (Figure 4A) and MEA paclitaxel dose trend (included in Supplementary Statistics File), the value of *k*_0.5_ assumed is not important. A different value of *k*_0.5_ will shift the complete bifurcation diagram, however, the qualitative structure of the diagram will remain the same.

The mathematical model that we considered is a minimal model representing dynamics of one type of peripheral neurons, putative small pain-sensing DRG neurons. This model provides a framework for future studies of other channels, pumps, and exchangers in hyperexcitability due to CIPN. *In vivo*, many more ion channels are present in these DRG neurons and they can be added to the model in future studies. For example, transient receptor potential channels (TRP) have also been suggested to be involved in CIPN (Hara et al., 2013; Chukyo et al., 2018). In addition, calcium channels can play a role in CIPN (Schmitt et al., 2018). These molecules will be excellent candidates for future studies, which may provide additional insights to CIPN. More detailed mathematical models have been developed previously and can be used for this purpose (Mandge and Manchanda, 2018). Paclitaxel can also induce cytosolic calcium oscillations (Boehmerle et al., 2006), which can again be analyzed using bifurcation theory. Another factor to add in future models is the impairment of axonal transport that occurs due to paclitaxel and its effect on microtubules (Nicolini et al., 2015), which can be modeled using cable equations (Holmes, 2013). In addition, a multicompartment modeling approach can be undertaken to investigate how spike propagation across the T-junction of the DRG neuron is regulated by the ion channels (Gemes et al., 2013; Du et al., 2014; Sundt et al., 2015). As mentioned previously, the effect of paclitaxel on ion channels may be indirect and due to other inflammatory cytokines (Aromolaran and Goldstein, 2017). In the future, this can also be included in the next phase of modeling. Lastly, our model consists of only small DRG neurons. However, paclitaxel impacts medium and large DRG neurons as well (Zhang and Dougherty, 2014). It will be of interest to evaluate models representing different subtypes of DRG neurons and investigate the role of differentially expressed channels specific to each subtype. The MEA experiment recorded firing of all DRG neuron subtypes. The blocker and enhancer used in the MEA experiments may have regulated ion channels besides those in small DRG neurons. Similarly, paclitaxel may have impacted the firing rate of different DRG neuron subtypes as well as other supporting cells such as glia. This will require further investigation in the future by recording DRG neuron subtype-specific firing rate. While additional compartments will be needed to make the model more comprehensive, its interpretation becomes challenging with increasing complexity. With the current model, it is difficult to explore how the MMOs arise due to the multiple time scales. A model dimension reduction method such as that mentioned in Rubin and Wechselberger (2007) will be required for an analytical investigation.

DRG neuron firing has been correlated with neuropathic pain *in vitro* (Yang et al., 2016, 2018), thus analyzing changes in firing patterns allows for a surrogate to study “pain in a dish.” Recent advances in neuroelectrophysiology technology have allowed for more temporal and spatial dynamic data collection on live cells. We chose a non-invasive method to measure the electrophysiological properties (spontaneous/non-evoked firing rate) of a network of neurons through microelectrode array (MEA) recording. Therefore, we used MEA to support our model outputs by recording the neuronal activity of the effect of paclitaxel on cultured DRG neurons, as shown in Figure 5. Although MEA cannot provide specific electrophysiological firing patterns of non-spontaneously firing neurons to detect bifurcation points *ex vivo*, this method was chosen because it is non-invasive, high-throughput, and can access neurons in their regular culture medium which can be more physiologically relevant. Even though a change in firing rate does not demonstrate that the system undergoes a bifurcation, one can use the change in the number of spontaneously firing neurons and a change in firing rate as an indicator of a qualitative change in the dynamics of the neurons. Using MEA, we can investigate how this changes upon introducing paclitaxel and the modulators. As seen in Table 2, the number of spontaneously firing neurons increases upon introducing paclitaxel and decreases upon introducing the modulators, indicating a qualitative change in the dynamics of an increasing number of neurons. Moreover, the number of neurons that spontaneously fired after treatment was significantly higher with paclitaxel treatment alone as compared to paclitaxel treatment together with modulators (Figure 5B). In this way, MEA can be indirectly used to support our bifurcation analysis findings. In the future, it will be of interest to explore a strict evidence for the bifurcations by recording dynamics of a single neuron. Moreover, it will also be of interest to match the spiking patterns from the model, in particular the mixed-mode oscillations, to the recordings from a single neuron.

Our bifurcation analysis identified that the maximal conductances of Na_v_1.8 and KDR are involved with the induction of spontaneous firing, associated with paclitaxel-induced hyperexcitability. The results from this model demonstrated that paclitaxel affects hyperexcitability in a concentration-dependent manner, and that Na_v_1.8 and KDR conductances can impact this hyperexcitability. We propose that one of the mechanisms behind PIPN is paclitaxel regulating expression of these ion channels—which are essential for hyperexcitability to occur—in a concentration-dependent matter, either directly or indirectly. This approach, along with patient-specific pharmacokinetics of paclitaxel, can be applied to the clinic by obtaining the patient's electrophysiological profile using patch clamp or MEA and by using those values as parameters in our model to determine the optimal dose of paclitaxel based on the individual, and for examining acute vs. chronic onset of PIPN. Moreover, treatments can be designed to specifically block Na_v_1.8 or enhance KDR conductance to reduce hyperexcitability caused by paclitaxel since we have identified the corresponding channels as key contributors to the hyperexcitability related to PIPN.

## Data Availability Statement

The XPP code for this model (with and without paclitaxel) can be found in the ModelDB database (http://modeldb.yale.edu/264591).

## Ethics Statement

The animal study was reviewed and approved by Institutional (Purdue) Animal Care and Use Committee.

## Author Contributions

PV, AK, DF, and DR designed and supervised the numerical bifurcation study. PV generated the bifurcation results. ME and YY designed the experimental study and ME performed the MEA experiments. PV and ME wrote the manuscript. Everyone participated in designing this study and reviewing the manuscript.

## Conflict of Interest

The authors declare that the research was conducted in the absence of any commercial or financial relationships that could be construed as a potential conflict of interest.
