# Supplementary material for: Examining Sodium and Potassium Channel Conductances Involved in Hyperexcitability of Chemotherapy-Induced Peripheral Neuropathy: A Mathematical and Cell Culture-Based Study
Source: Front Comput Neurosci. 2020 Oct 15;14:564980. doi: 10.3389/fncom.2020.564980 (PMC7593680; doi:10.3389/fncom.2020.564980)
Supplement: Supplementary file 2 [file Data_Sheet_1.PDF]

Supplementary Material

1 SUPPLEMENTARY DATA

Statistical summary for MEA analysis (included separately as an Excel sheet).

2 SUPPLEMENTARY FIGURES

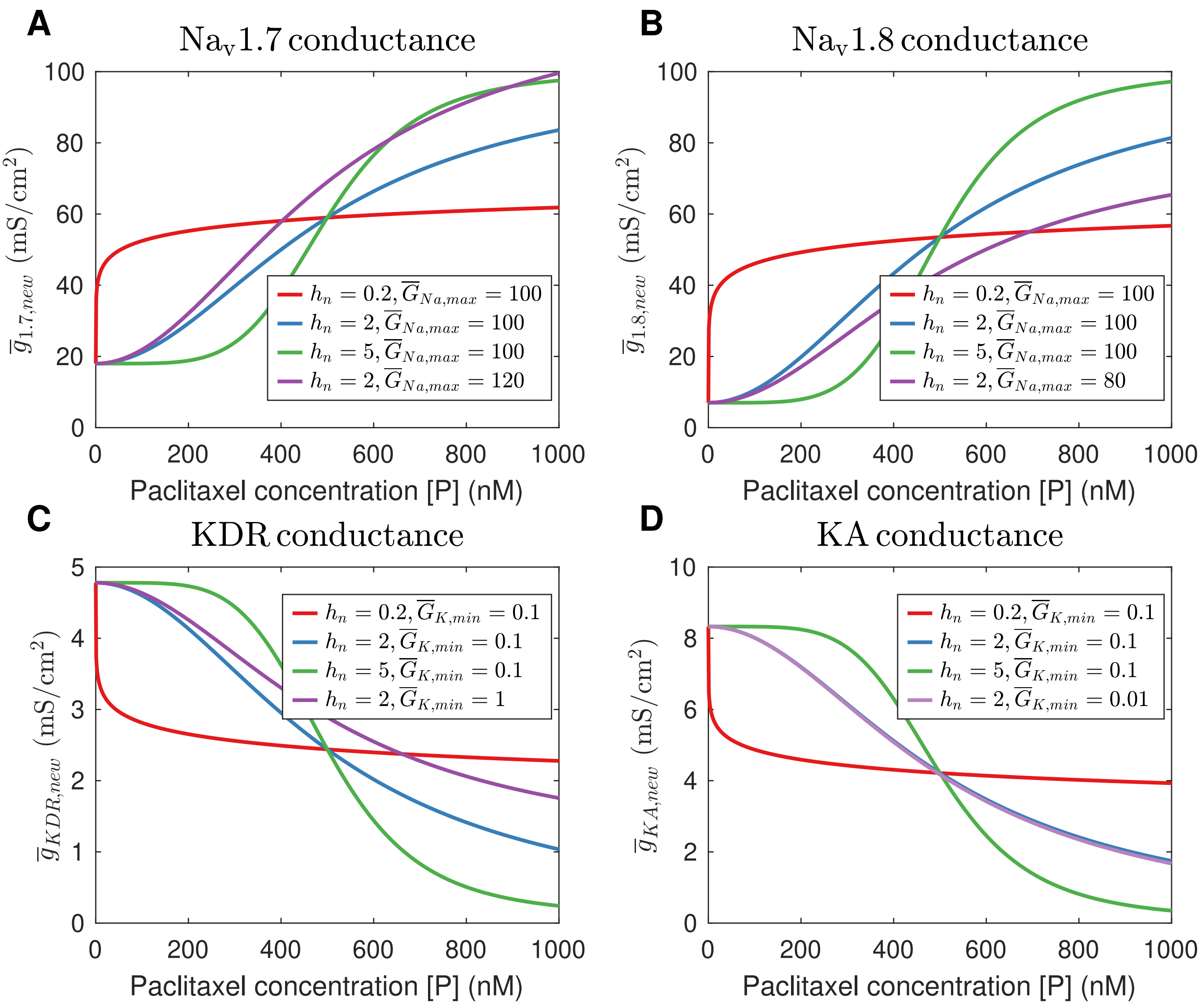

Figure S1. Effect of paclitaxel on conductances and firing. (A): Effect of paclitaxel on $\overline{g}_{1.7,new}$ upon varying h_n and $\overline{G}_{Na,max}$. Increasing $\overline{G}_{Na,max}$ will widen the parameter range of $\overline{g}_{1.7,new}$. Increasing h_n alters the curve to become more sigmoidal. (B): Similar effect is seen with $\overline{g}_{1.8,new}$. Decreasing $\overline{G}_{Na,max}$ will reduce the parameter range of $\overline{g}_{1.8,new}$. (C): Increasing paclitaxel concentration decreases $\overline{g}_{KDR,new}$. As before, increasing h_n makes the curve more sigmoidal. Decreasing $\overline{G}_{K,min}$ increases the parameter range. (D): Similar effect is seen for $\overline{g}_{KA,new}$. Increasing $\overline{G}_{K,min}$ decreases the parameter range in this case. The blue curves correspond to the parameter values that were considered for bifurcation analysis in the main manuscript. Note that the blue and purple curves are overlapping in D, thus the blue curve is not visible.

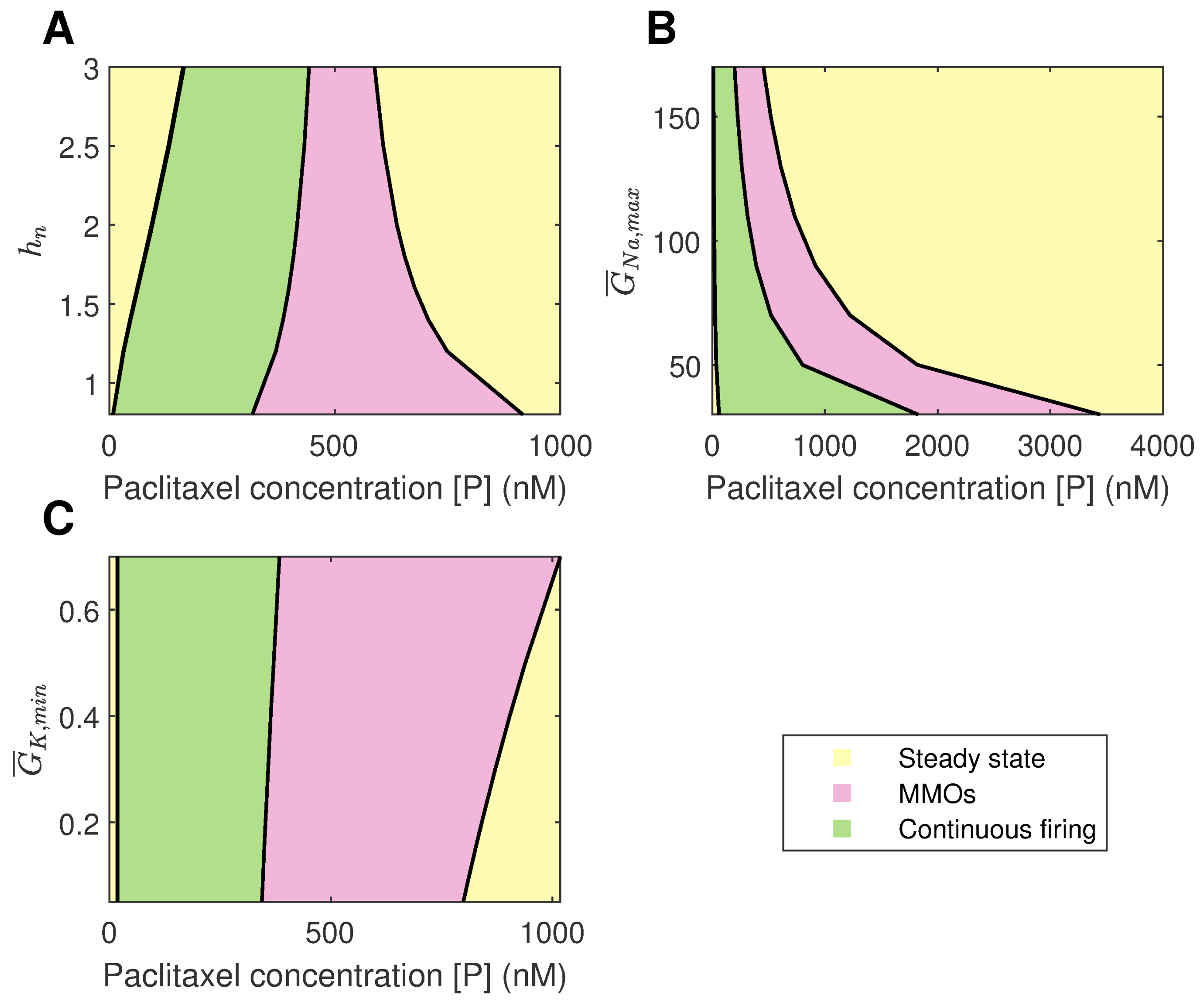

Figure S2. Regions of stable steady state, MMOs, and continuous firing upon varying h_n, $\overline{G}_{Na,max}$, and $\overline{G}_{K,min}$ with paclitaxel concentration [P]. (A): Continuation of Hill's coefficient h_n. Upon increasing h_n, the spontaneous firing regime becomes narrower. (B): Continuation of $\overline{G}_{Na,max}$. Upon increasing $\overline{G}_{Na,max}$, the spontaneous firing regime becomes narrower. (C): Continuation of $\overline{G}_{K,min}$. Upon increasing $\overline{G}_{K,min}$, the spontaneous firing regime becomes wider.
